# Mutations in *FKBP10 *can cause a severe form of isolated Osteogenesis imperfecta

**DOI:** 10.1186/1471-2350-12-152

**Published:** 2011-11-22

**Authors:** Ortrud K Steinlein, Eric Aichinger, Holger Trucks, Thomas Sander

**Affiliations:** 1 Institute of Human Genetics, University Hospital, University of Munich, Munich, Germany; 2 Cologne Center for Genomics, University of Cologne, Cologne, Germany

## Abstract

**Background:**

Mutations in the *FKBP10 *gene were first described in patients with Osteogenesis imperfecta type III. Two follow up reports found *FKBP10 *mutations to be associated with Bruck syndrome type 1, a rare disorder characterized by congenital contractures and bone fragility. This raised the question if the patients in the first report indeed had isolated Osteogenesis imperfecta or if Bruck syndrome would have been the better diagnosis.

**Methods:**

The patients described here are affected by severe autosomal recessive Osteogenesis imperfecta without contractures.

**Results:**

Homozygosity mapping identified *FKBP10 *as a candidate gene, and sequencing revealed a base pair exchange that causes a C-terminal premature stop codon in this gene.

**Conclusions:**

Our study demonstrates that *FKBP10 *mutations not only cause Bruck syndrome or Osteogenesis imperfecta type III but can result in a severe type of isolated Osteogenesis imperfecta type IV with prenatal onset. Furthermore, it adds dentinogenesis imperfecta to the spectrum of clinical symptoms associated with *FKBP10 *mutations.

## Background

Osteogenesis imperfecta (OI) is a rare monogenic disorder that shows both clinical and genetic heterogeneity. The disorder is characterized by bone fragility that varies in severity between the different OI subtypes. In the majority of patients the disorder is caused by dominant mutations in the procollagen genes *COL1A1 *and *COL1A2 *[1-4]. Even rarer than the dominant disorders are the autosomal recessive forms of OI for which several genes are known by now. These genes are mainly involved in the processing, assembly and trafficking of procollagen chains, a multi-step process that involves a large number of critical post-translational modifications. Homozygosity for mutations in *CRTAP*, *LEPRE1*, *PPIB*, or *OSTERIX *has been shown to cause mostly severe types of OI that result from overmodification of procollagen chains and their retention in the rER [2-7]. Autosomal recessive brittle bone disease without procollagen overmodification can be caused by mutations in either *SERPINH1 *or *FKBP10 *genes that encode the collagen chaperone-like proteins HSP47 and FKBP65, respectively [1,3]. Basic pathomechanisms of OI initiated by mutations in these genes might include a failure to mature type I procollagen trimers and increased protease sensitivity [3,8]. The pathomechanisms of another OI gene, *SERPINF1*, are still unknown [9]. Mutations in *FKBP10 *were first described in patients with a moderate to moderate/severe form of OI type IV (OMIM 610968) [1]. Two follow up reports presented patients in which *FKBP10 *mutations were associated with Bruck syndrome type 1, a rare disorder characterized by congenital contractures and bone fragility [10-12]. The question was raised if the patients in the first report indeed had isolated OI or if Bruck syndrome would have been the better diagnosis [11,12]. We here describe three patients showing that *FKBP10 *mutations not only can cause isolated OI but that the spectrum of clinical symptoms is much broader than previously described.

## Methods

### Patients

The three affected brothers, their sister and their parents provided informed consent for molecular testing, clinical assessment and the publication of their photographs. Anonymous blood samples from 80 healthy German medical students were used as controls. The experiments were performed in accordance to the Helsinki Declaration and approved by the Ethics committee of the University of Munich.

### Homozygosity mapping and FKBP10 sequencing

Genomic DNA samples of family members I1-2, II1-2, and II4-5 were genotyped using the Affymetrix Axiom™ Genome-Wide Human Array, featuring 567,096 SNP markers (Affymetrix, Santa Clara, CA, USA). Extended runs of homozygosity (ROHs) shared by the three affected brothers were assessed in comparison to the unaffected family members using the program HomozygosityMapper (http://www.homozygositymapper.org) [13]. Prioritization of candidate genes within the homozygous region was performed by the program GeneDistiller (http://www.genedistiller.org) [14] and by comparison to PubMed entries (http://www.ncbi.nlm.nih.gov/pubmed). The complete coding sequence of the *FKBP10 *gene was amplified and directly sequenced.

## Results

### Clinical findings

**Figure 1:**
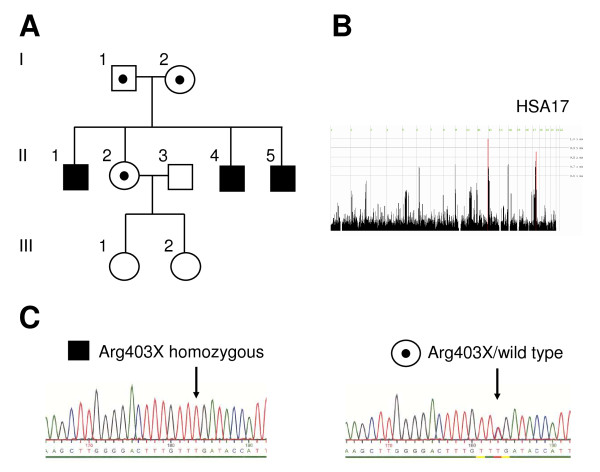
**Genetic studies.** A, Pedigree of the OI family. Black pedigree symbols, affected brothers, homozygous; symbols marked by black dots, unaffected family members that are heterozygous for the mutation; unmarked symbols, unaffected family members that were not available for analysis. B, Genome wide plot of Homozygosity Mapper. Red bars indicate regions with extended ROH at chromosomal regions 11q25 and 17q21.2, shared by the three affected brothers. *Homozygosity Score *threshold 0.8; HSA, *homo sapiens *chromosome. C, Sequencing results. Parts of *FKBP10 *exon 7 are shown. Left side, OI patient II1 homozygous for Arg403X; right side, healthy sister (II2) heterozygous for Arg403X.

**Figure 2:**
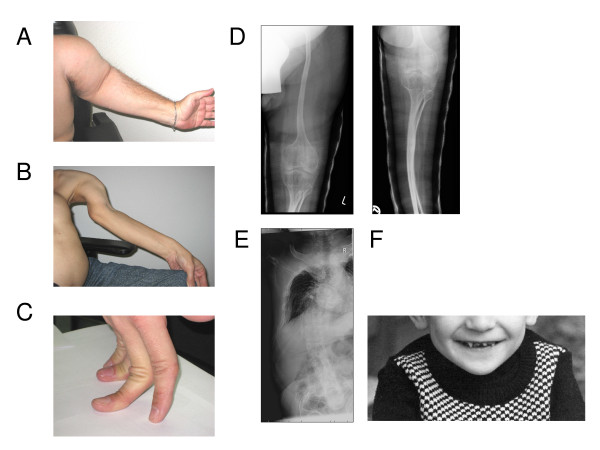
**Clinical phenotype**. A-B, extended left arm II1, II5. Upper arm is severely shortened in II1 and instabilized by pseudarthrosis in II5. Webbing and contractures are absent; C, joint hypermobility, demonstrated by II4; D, Radiographs of left leg and E, left side of thorax showing severe kyphoscoliosis (II4); F, Dentiogenesis imperfecta (primary teeth) in II5.

The three affected brothers, aged 46 (II1), 44 (II4), and 43 (II5) years, were born to healthy, unrelated parents. There is no family history of bone diseases or major malformations. The oldest brother was born without fractures but showed delayed motor development during his first year of life. He had his first fracture at age 9 months. The second brother was born with multiple fractures. Multiple fractures of the upper extremities at birth were also suspected in the youngest brother but not confirmed by X-ray. So far each of the brothers already had 70-100 fractures and breaks still occur. Severe progressive kyphoscoliosis has caused reduced lung capacity in all three brothers, and they are wheel-chair bound due to long bone deformities. None of the brothers showed contractures or webbing at birth. The brothers are of short stature (120-140 cm, <3 %tile) with remarkable hypermobile joints (Figure 1). Relevant lab results such as alkaline phosphatase (73 U/l) are within the normal range. The primary teeth were discoloured and rapidly wore down, thus showing typical signs of dentinogenesis imperfecta (Figure 2). The permanent teeth are less affected. Blue sclera were noted in childhood but are no longer present.

### Genetic studies

Homozygosity mapping detected two candidate ROHs in the chromosomal regions 11q25 and 17q21, respectively (Figure 1B). The ROH on 17q21.2 (17: 39727018-44200191, rs1010594-rs9910284, NCBI Genome Build 37.3) harbors the *FKBP10 *gene encoding the FKBP65 protein (Additional File 1, Figure S1). Sequencing revealed a homozygous nucleotide exchange in exon 7 that changes an arginine codon into a stop codon (c.1207C>T, FKBP65-p.(Arg403*) (Figure 1C).

## Discussion

We identified a homozygous C-terminal premature stop codon (FKBP65-p.Arg403X) in the *FKBP10 *gene causing OI in three affected siblings of a family with autosomal recessive inheritance. Both parents derived from two neighbouring small villages in Bavaria, suggesting a restricted genetic variability and a potential accumulation of recessive founder mutations in this rather isolated population. Although estimates of the relative relationship by identity-by-state allele sharing did not indicate consanguinity (see Additional File 1), homozygosity mapping was successfully applied to identify a causative homozygous *FKBP10 *founder mutation. Our study demonstrate that homozygosity mapping represents a suitable diagnostic approach to map recessive OI genes even in outbred populations [15].

The first *FKBP10 *mutations were found in families that were described as having OI but for which no contractures or webbing were reported [1]. Shaheen et al. reviewed the figures in the original publication and questioned the absence of webbing [11,12]. Both contractures and webbing were present in their own patients as well as in all but two of the patients reported by Kelley et al.[10] The patients were therefore diagnosed with Bruck syndrome type 1, a distinct form of OI that is characterized by brittle bone disease and arthrogryposis multiplex congenita. Interestingly, case 2 and 4 in the report from Kelley et al. had OI but did not fulfil the criteria for Bruck syndrome [10]. In our family none of the three brothers had any contractures at birth or showed webbing. Thus, taken together, the previously reported patients and the family described here demonstrate that *FKBP10 *can cause either Bruck syndrome or isolated OI. Additional families need to be collected to answer the question if these two clinical phenotypes are associated with specific mutations, or if isolated OI and Bruck syndrome can occur in the same family.

So far it is not known if the severity of the disorder is correlated to the position of the mutation within the *FKBP10 *gene. Most patients carry frame shifting mutations that are located in the N-terminal half of the gene and are likely to cause nonsense mediated decay (NMD) (1). Interestingly, the affected brothers described here are homozygous for a C-terminal premature stop codon that, according to its vicinity to intron 7, might not initiate NMD [16,17]. The resulting truncated protein would still have the protein-binding PPIase domains that are necessary for its chaperone function but would miss the putative rER-retention sequence (HEEL). The chaperone function might have destructive effects if exhibited outside the rER.

With the exception of some of the patients described by Alanay et al. [1] most previously published patients with *FKBP10 *mutations had a moderate to moderate-severe form of the disorder that is best classified as OI type III. The data summarized in Table 1 show that fractures mostly started a few weeks after birth, sometimes even later, but not pre- or perinatally [1,10-12]. Progressive skeletal deformities were rarely described. In our family at least one, but more likely two of the affected brothers were already born with multiple fractures. Subsequently, all three brothers developed extreme deformities of their long bone and spines, fulfilling the criteria for severe OI (Figure 2). Furthermore, dentinogenesis imperfecta was present in all three brothers but has so far not been described as a feature of patients with *FKBP10 *mutations [1,10-12]. The combination of symptoms in our family does not match the classification of OI type III but rather suggests that this family belongs to the subgroup of patients with OI type IV that show progressive skeletal deformities and dentinogenesis imperfecta.

**Table 1:** Summary of known FKBP10 mutations

Mutations in OI and Brucks syndrome patients	Type	Position	OI severity	Reference
c.117_152del (p.Leu41GlnfsX22) homozygous	frame shift deletion	upstream of 1st PPIase domain	Fractures beginning at age 3 years	[13]
c.321_353del (p.Gly107-Leu117del) homozygous	in frame deletion	1st PPIase domain	Fractures beginning in infancy	[11]
c.321_353del (p.Met107-Leu117del*)/c.1276-_1277insC (p.Glu426ProfsX479) heterozygous	in frame deletion/frame shift insertion	1st PPIase domain/HEEL domain	nm	[13]
c.344G>A, (p.Arg115Gln)/c.831dupC (p.Gly278ArgfsX295) heterozygous	frame shift insertion/missense mutation	1st PPIase domain/	Fractures beginning in infancy (fist weeks of life)	[13]
c.831dupC* (p.Gly278ArgfsX295) homozygous	frame shift duplication	between 2nd and 3rd PPIase domain	Fractures beginning in infancy (2 months of age)	[11] [13]
c.1016_1023dup (p.Thr342GlyfsX26)	frame shift duplication	3^rd ^PPIase domain	Fractures beginning in infancy (7 months of age)	[14]
c.1207C>T (R403X) homozygous	nonsense mutation	HEEL domain	Multiple fractures in fetal period	Family presented here

Predicted amino acid mutations are given in brackets; *mutation nomenclature has been adapted to match the entries in the Osteogenesis Imperfecta Variant Database (https://oi.gene.le.ac.uk);

## Conclusions

Taken together, our study shows that a severe, progressive course of OI type IV with pre- or perinatal onset of fractures and dentinogenesis imperfecta has to be added to the spectrum of clinical features that can be associated with *FKBP10 *mutations. We also demonstrate that homozygosity mapping represents a suitable diagnostic approach to map recessive OI genes even in outbred populations.

## Abbreviations

*FKBP10*: FK506-binding protein; HEEL: rER-retention sequence OI; osteogenesis imperfecta; NMD: nonsense mediated decay; rER: rough endoplasmatic reticulum; ROHs: runs of homozygosity.

## competing interests

The authors declare that they have no competing interests.

## Authors' contributions

OST planed the molecular genetic studies, collected the clinical data and drafted the manuscript. EA participated in the clinical and molecular part of the study. HT performed the homozygosity mapping. TS participated in the study' design and carried out the statistical analysis. All authors read and approved the final manuscript.

## Pre-publication history

The pre-publication history for this paper can be accessed here:

http://www.biomedcentral.com/1471-2350/12/152/prepub

## Supplementary Material

Additional file 1 **Supplementary Figure S1: Runs of homozygosity at 17q21.2**. Supplementary Figure S2: Graphical relative relationship of individuals with different degrees of relationship. Click here for file
