# Supplementary material for: Mutations in FKBP10 can cause a severe form of isolated Osteogenesis imperfecta
Source: BMC Med Genet. 2011 Nov 22;12:152. doi: 10.1186/1471-2350-12-152 (PMC3270005; doi:10.1186/1471-2350-12-152)
Supplement: Additional file 1 — Supplementary Figure S1: Runs of homozygosity at 17q21.2. Supplementary Figure S2: Graphical relative relationship of individuals with different degrees of relationship. [file 1471-2350-12-152-S1.DOC]

**Supplement for:**

**Mutations in *FKBP10* can cause a severe form of isolated osteogenesis imperfecta**

Ortrud Steinlein ^1^, Eric Aichinger ^1^, Holger Trucks ^2^, Thomas Sander ^2^

^1^Institute of Human Genetics, University Hospital, University of Munich, Munich, Germany, Ortrud.Steinlein@med.uni-muenchen.de, Eric.Aichinger@med.uni-muenchen.de

^2^Cologne Center for Genomics, University of Cologne, Cologne, Germany, Holger.Trucks@uni-koeln.de, Sandert@uni-koeln.de

Address for correspondence and reprints:

Ortrud K. Steinlein, M.D., Ph.D.

Institute of Human Genetics

University Hospital

University of Munich

Goethestr. 29 tel: (49 89) 5160-4470

80336 Munich fax: (49 89) 5160-4468

Germany [Ortrud.Steinlein@med.uni-muenchen.de](mailto:Ortrud.Steinlein@med.uni-muenchen.de)

**Supplementary Figure 1:** Runs of homozygosity at 17q21.2


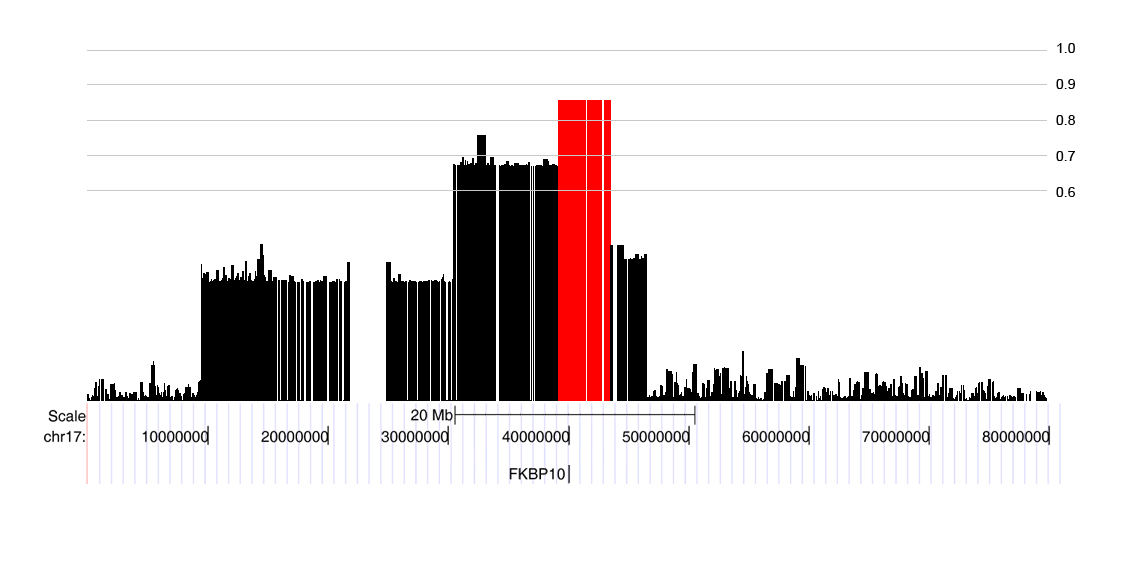


**Legend:** The red bar indicates a 4.5 Mb region at 17q21.2 showing a run of homozygosity consisting of 285 consecutive single nucleotide polymorphisms shared by the three brothers affected by osteogenesis imperfecta. The x-axis represents the physical position of nucleotides along chromosome 17 (NCBI Build 37.3) and indicates the position of the *FKBP10* gene.

**Supplementary Figure 2:** Graphical relative relationship of individuals with different degrees of relationship


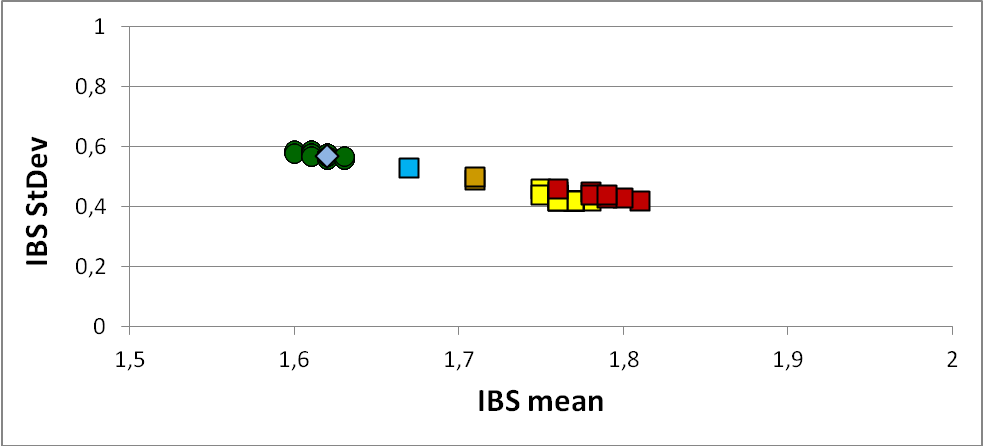


**Legend:** Identity-by-state (IBS) allele sharing of all individual pairs was determined by the GRR software (http://bioinformatics.well.ox.ac.uk/GRR). The parent-pair of the osteogenesis imperfecta family is shown as diamond in light blue colour, indicating a similar IBS allele sharing like those of sixteen unrelated parents from the same ethnic origin (green circles). In comparison IBS allele sharing is shown for different degrees of relationship: a) first-degree relationship of parent-offspring pairs (yellow squares); b) second-degree relationship of sib-pairs (red squares); c) third-degree relationship (brown squares); d) fourth-degree relationship (blue square); e) unrelated parents of an outbred population (green circles).
